# Investigating human exposure to a practical wireless power transfer system using and the effect about key parameters of dosimetry

**DOI:** 10.1371/journal.pone.0236929

**Published:** 2020-08-14

**Authors:** SangWook Park

**Affiliations:** Division of Electronic & Electrical Engineering, College of Information and Communication Engineering, Daegu University, Gyeongsan, Korea; University of Glasgow, UNITED KINGDOM

## Abstract

Accurate dosimetry for a real wireless power transfer system (WPT) using electromagnetic resonance and electromagnetic induction requires an accurate description of the field formed by the system. In particular, the electromagnetic field depends on factors such as the construction of the transmitting and receiving coils, the circuit configuration, the input source of the front end of the transmitting coil, and the input impedance of the rear end of receiving coil. However, both circuit and electromagnetic simulations need to be performed to analyze the entire system, which is a difficult task. In order to overcome this difficulty, a method using an equivalent circuit model is proposed and verified through experiments. Moreover, the worst exposure condition to a magnetic field was examined by considering three variables: the charging mode, the state of charge, and the alignment and misalignment between the transmitting and receiving coils. Accordingly, the strongest magnetic field was created in the constant current mode in the fully charged state with misalignment. For example, the magnetic field strength in the case of 80% state of charge and misalignment was 1.397 times greater than in the case of 20% state of charge and alignment at a point 10 mm from the transmission pad. Finally, the induced electric fields and induced current densities were calculated by using a Japanese adult male whole-body voxel human model, and the results were compared with the values recommended by international guidelines to ascertain their compliance.

## Introduction

Many studies have been focused on the application of WPT technology to various fields [1–4]. Some technical issues such as the physical understanding of WPT, design theory, matching techniques for achieving a high-power transfer efficiency, and the improvement in the power transfer distance, have almost all been resolved. WPT emits more electromagnetic energy than wireless communication [5, 6]. Thus, people are more concerned about and interested in human exposure to electromagnetic radiation. This problem is one of the last hurdles for electromagnetic compatibility and standards for commercialization. In particular, as the application of this technology to electric vehicles, trains, etc. will be accompanied by high-power transfer, the electromagnetic effects on the human body should be carefully examined.

Guidelines for human protection from electromagnetic field exposure are provided by either the International Commission on Non-Ionizing Radiation Protection (ICNIRP) [7, 8] or IEEE safety standard [9, 10]. The risk assessment for a human body exposed to a WPT system requires the calculation of the induced quantities in the human body, and compliance of the result with chosen international guidelines is to be checked. WPT systems using electromagnetic induction and resonance are one of the most actively studied subjects, and the operating frequency of these systems is in the range of frequency bands on the order of hundreds of kilohertz or several megahertz. Thus, an investigation should focus on the use of the operating frequency band below dozens of megahertz for a WPT system. As the induced quantities in a human body are difficult to measure in such a frequency band, they have been calculated by a numerical analysis using a human body model [11–24]. This numerical dosimetry can be performed successfully under the condition that the electromagnetic field to which a human body will be exposed is accurately simulated. This condition is met only by accurately describing the current flowing in a WPT coil. However, the current depends on the terminal condition, the state of charge (SoC), and so on (details will be presented in Section II-A). Thus, a simulation needs to be adapted to the source and load conditions that vary according to the SoC. In this regard, it is very important to perform experimental verification or to present information about the port condition. Otherwise, dosimetry could be carried out under an abnormal condition that fails to reflect a real system.

To the best of our knowledge, from early studies to more recent ones [11, 12, 14, 18, 19] the magnitudes of the electromagnetic fields created by a WPT system have been obtained only by simulations without experimental verification, and the simulated fields have been used for numerical dosimetry as an incident field. Most early studies performed dosimetry by setting the impedance to 50 Ω on both the source and load sides and the input power to 1 W. Some studies evaluated the power transfer efficiency by the square of the transmission coefficient (S21) of the scattering parameters. However, this calculation is limited to the case where the ports on both sides are terminated by 50 Ω [13]. The power transfer efficiency should be calculated by using the voltages and currents on both sides of the coil, thus using the ratio between the input and output powers. The authors of [11] verified the simulated electromagnetic field by measurement, but dosimetry was performed only with a 50-Ω port. In [12], the simulated results for the magnetic field and the current in some coils are compared with the experimental results of a paper written in Japanese [20], but no information about the real source and load conditions is presented. In [16] and [17], experimental verification and numerical dosimetry were carried out for low-power WPT systems such as mobile phones, which were specified in the Wireless Power Consortium (WPC) standard for the 100-kHz band and in the AirFuel Alliance standard for the 6.78-MHz band. However, these studies also lack accurate descriptions of the termination conditions of the load and source. Recently, in [21], a study was conducted to measure the magnetic field from an actual wireless charging system and to simulate the value as an incident field of a human body model. Except for this method, in order to obtain the value of the incident field through the simulation, the simulation must be performed exactly under the same conditions as in the real world. In [22], the simulation technique using the equivalent circuit is applied, but verification by comparison between the measured value and the simulated value regarding the magnetic field has not been performed and there is no detail explanation of how to use an equivalent circuit.

**Fig 1:**
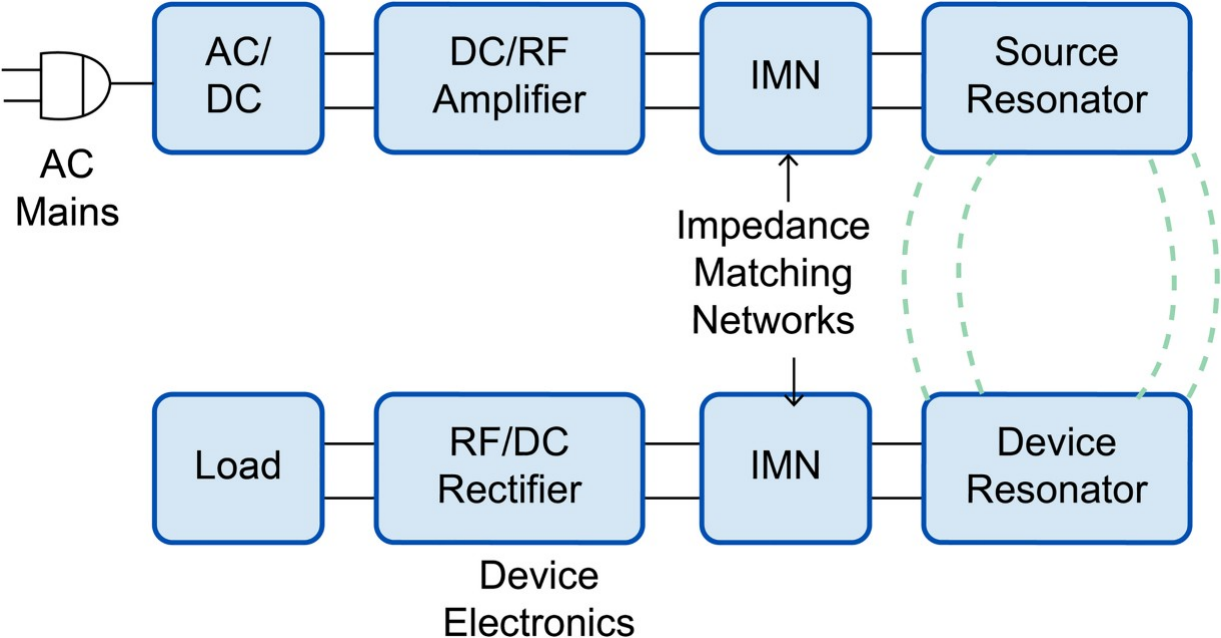
Block diagram of a WPT system (source: [26]).

As shown in Fig 1, in a practical WPT system the transmitting and receiving coils are connected to various subcircuits, such as inverter and rectifier and the ports of the coils cannot be simply equalized to 50 ohms [23, 25]. Therefore, the currents flowing in the transmitting and the receiving coils in a practical system differ significantly from those in a system simply terminated at 50 ohms. For example, when 3.3 kW of power is transmitted by the model used in this study, the current flowing through the transmitting coil is approximately 24 A (rms); by contrast, when the transmission coil and receiving coils are terminated by 50 ohms, the current is approximately 11 A (rms), a 54% difference in current. Because the magnitude of the external magnetic field formed around the wireless charging system is proportional to the magnitude of the current flowing through the coil, the difference in current results in a proportional difference in the magnitude of the field. The induced current density (or internal electric field) within the human body is directly proportional to the external magnetic field and the specific absorption rate (SAR) is proportional to its square. Thus, the induced values of these factors are underestimated by 54 and 79%, respectively, under the 50-ohm case. This cascade of modeling errors underscores the necessity of accurately describing the current flowing in the transmitting and receiving coils of the charging system.

Consequently, it is of utmost importance to describe the field accurately. Electromagnetic simulation, which includes various circuits and active elements and is created for the transmitting and receiving coils, is almost impossible. Thus, a simulation technique that can separate the transmitting and receiving coil section of interest from the entire circuit configuration of the WPT system and model the section as a real system is needed.

In Section II, the theory related to the variation in the electromagnetic field according to the SoC (change in load) in a WPT system for an electric vehicle is discussed. In Section III, the experimental results for verification and an equivalent circuit method proposed to accurately simulate the conditions of a real WPT system are presented. In addition, to identify the worst exposure scenario, the SoC, the charging mode (constant current, power, and voltage), and the offset of the transmitting and receiving coils are investigated, which are variables affecting the electromagnetic fields around a WPT system. Finally, in Section IV, three conditions are set, which allow the changes in the electromagnetic field caused by these variables to be compared, and dosimetry is performed for these conditions.

## The worst exposure and fabricated WPT system

### Power transfer process and the worst exposure scenario

When a magnetic field radiated from a WPT system for electric vehicles is measured to ensure the protection of a human body from electromagnetic waves, clear identification of the worst possible situation is crucial for an accurate assessment of exposure. The magnetic field around a WPT system depends on the currents flowing in the transmitting and receiving coils. Although there are many variables affecting the human exposure to electromagnetic waves, we choose three key issues, assuming that the received power is fixed. They are (i) the charging mode, (ii) the SoC, and (iii) the alignment status of the transmitting and receiving coils. This section is devoted to an analysis of these three variables along with an explanation of the power transfer process in electric vehicles.

ISO 15118 [27] is the standard for the wired charging process of electric vehicles. WPT is expected to be specified according to the same standardization system. The battery management system (BMS), which is mounted on an electric vehicle, controls the power transfer to ensure the safe management of the battery. In other words, the amount of current required to charge the battery of an electric vehicle is not determined by the charger. Rather, the BMS determines the required current and demands it from the charger.

#### (i) Charging mode

Power transfer typically starts in the CC or constant power (CP) mode, which maintains a constant level of current and power, respectively. Near the end of the process, the mode is changed to the constant voltage (CV) mode in order to maintain the specified battery voltage in the fully charged state. Such a charging mode can change the current flowing in the transmitting and receiving coils.

#### (ii) State of charge

**Fig 2:**
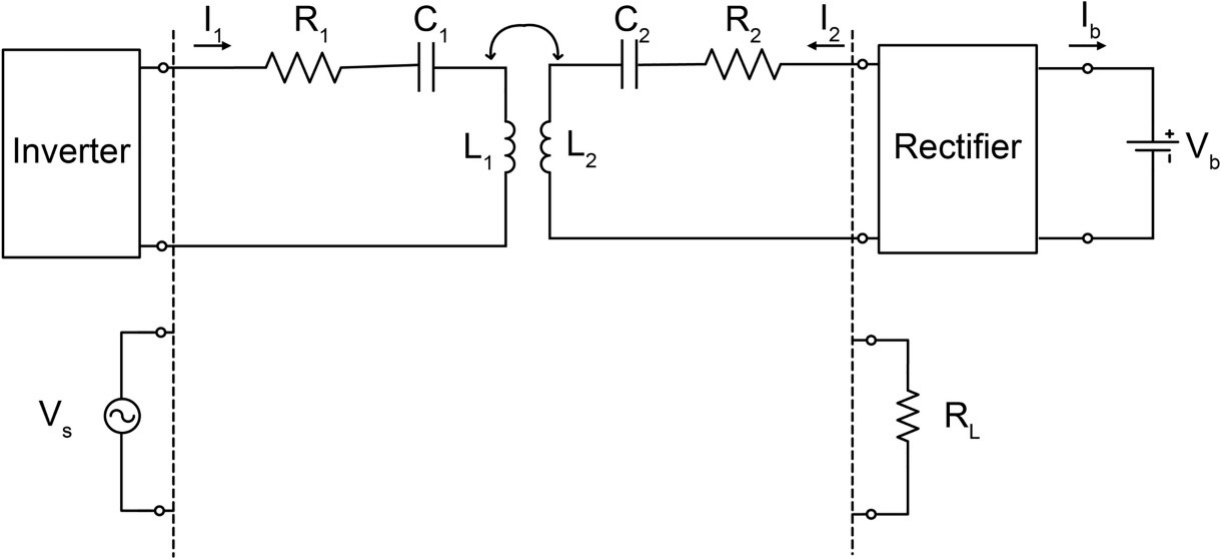
Block diagram and equivalent circuit of the WPT system.

For the sake of illustration, we examine the equivalent circuit of the resonance part, as shown in Fig 2, which is the most basic and most critical element. The following formula is obtained by applying Kirchhoff’s current law (KCL) to the closed loops on both sides:

$$\left[\begin{array}{c} V_{s}\\ 0 \end{array}\right]=\left[\begin{array}{cc} R_{1}+j(\omega L_{1}-\frac{1}{\omega C_{1}}) & -j\omega M\\ -j\omega M & R_{2}+j(\omega L_{2}-\frac{1}{\omega C_{2}})+R_{L} \end{array}\right]\left[\begin{array}{c} I_{1}\\ I_{2} \end{array}\right],$$ (1)

where *R*_1_ and *R*_2_ are the resistances of the coils, *L*_1_ and *L*_2_ are the inductances of the coils, *C*_1_ and *C*_2_ are the capacitances added to generate resonance at the desired frequency, and *M* is the mutual inductance between two coils. The currents in the transmitting and receiving coils can be obtained as follows:

$$\left[\begin{array}{c} I_{1}\\ I_{2} \end{array}\right]=\frac{V_{s}}{(R_{1}+j(\omega L_{1}-\frac{1}{\omega C_{1}}))(R_{2}+j(\omega L_{2}-\frac{1}{\omega C_{2}})+R_{L})+{\left(\omega M\right)}^{2}}\times [\begin{array}{c} R_{2}+j(\omega L_{2}-\frac{1}{\omega C_{2}})+R_{L}\\ j\omega M \end{array}].$$ (2)

The transmitting power or input power at the transmitting coil is obtained by the following formula:

$$P_{in}=Re\left\{V_{S}I^{*}{}_{1}\right\}/2,$$ (3)

where *V*_s_ and *I*_1_ are the excitation voltage and the current at the input port of transmitting coil, respectively, and * represents the conjugate. The receiving power or output power at the battery is directly obtained by the following formula:

$$P_{out}=I_{b}V_{b},$$ (4)

where *V*_b_ and *I*_b_ are the battery voltage and the current flowing into the battery, respectively. Here, the power transfer efficiency is defined by:

$$\eta =\left(P_{out}/P_{in}\right)\times 100.\;\left(\%\right)$$ (5)

If the subcircuit that is seen towards the rectifier and battery can be expressed by the equivalent resistance *R*_L_, the output power is calculated by the current at the load part by:

$$P_{out}=R_{L}|I_{2}{|}^{2}/2$$ (6)

where *I*_2_ is the current at the load port. *R*_L_, which varies according to the SoC, can be produced by measuring *P*_out_ and *I*_2_ and then using (6).

The battery voltage changes according to the SoC. In other words, as power transfer proceeds, the battery voltage increases. When the voltage at *R*_L_ is *V*_2_,

$$V_{2}=-I_{2}R_{L},$$ (7)

and if (1) and (7) are expressed as a relation between the voltage and the current, the following formulas can be obtained:

$$V_{s}=\left\{R_{1}+j\left(\omega L_{1}-\frac{1}{\omega C_{1}}\right)\right\}I_{1}-j\omega MI_{2},$$ (8)

$$V_{2}=\left\{R_{2}+j\left(\omega L_{2}-\frac{1}{\omega C_{2}}\right)\right\}I_{2}-j\omega MI_{1}.$$ (9)

If the resistance of a coil is neglected and a resonance frequency is applied, (8) and (9) can be simplified as follows:

$$V_{s}=-j\omega MI_{2},$$ (10)

$$V_{2}=-j\omega MI_{1},$$ (11)

From these formulas, the current flowing in the receiving coil is proportional to *V*_s_, and the current flowing in the transmitting coil is proportional to *V*_2_, which is related to the battery voltage. Consequently, the variation in the battery voltage leads to a change in the current in the receiving coil.

Given an input current *I*_2_ and input voltage *V*_2_ at the port looking into the rectifier and battery, this subcircuit can be replaced by the equivalent resistor *R*_*L*_. When an input current *I*_2_ determined by the source voltage *V*_*s*_ is applied to this sub circuit, the voltage of port 2 *V*_2_ also increases with the rise in battery voltage over time. To find the transient response of this battery-containing subcircuit, it would be necessary to construct a sophisticated equivalent model using the various models discussed in [28]. However, the goal here is to obtain the external magnetic field produced by the wireless charging system based on the precise determination of the current flowing under the application of a specific battery voltage and frequency. In this case, the specific battery voltage determined by the SoC and the current *I*_2_ determined by source voltage *V*_*s*_ are used to derive the equivalent resistance *R*_*L*_.

#### (iii) Alignment and misalignment between the transmitting and receiving coils

**Fig 3:**
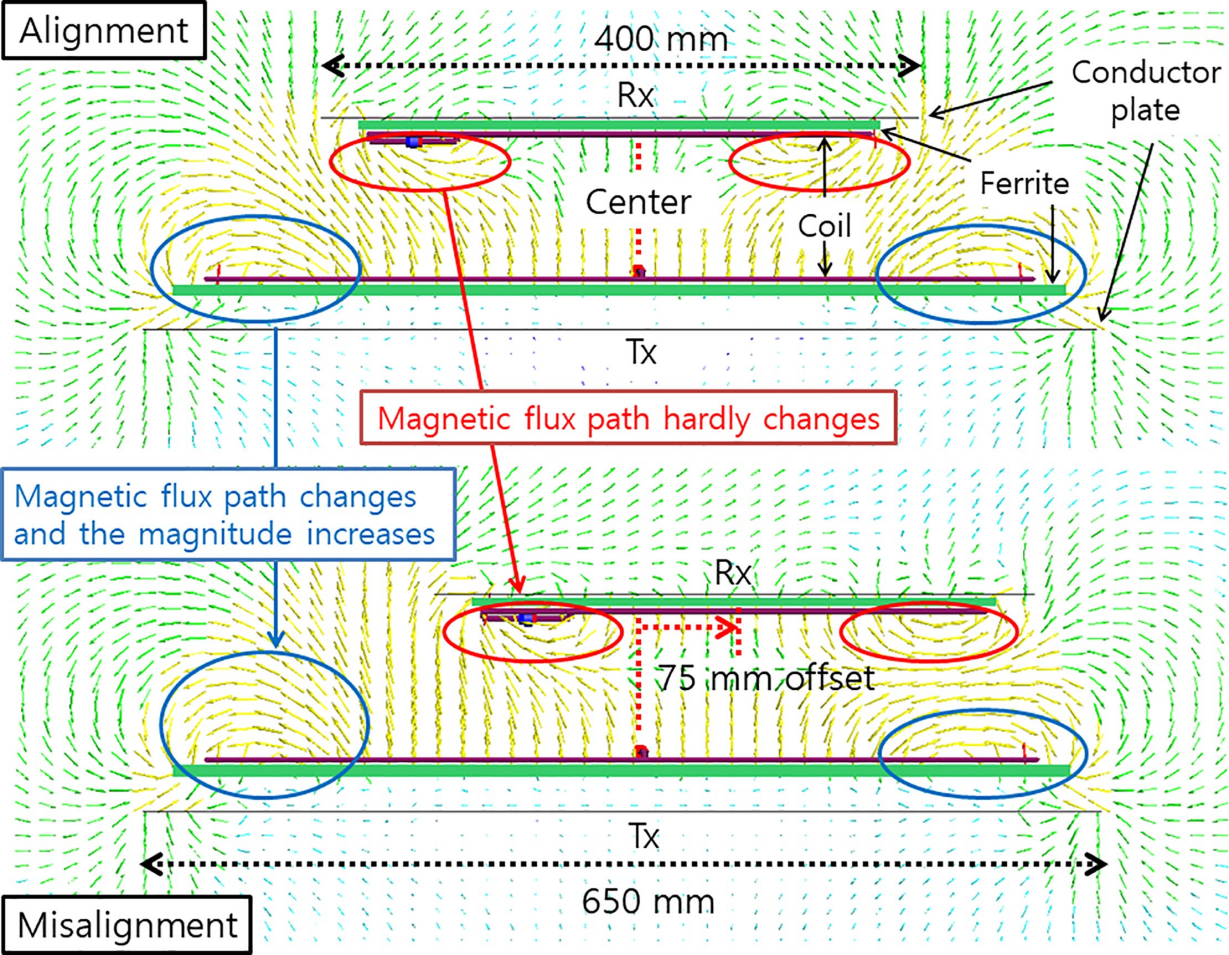
Magnetic flux distributions under alignment and misalignment.

We often encounter an exposure scenario in a misalignment situation that exists as an electric vehicle approaches a charging pad. Thus, this misalignment exposure scenario will be considered in this subsection. The misalignment between the transmitting and receiving coils decreases *M*, thereby degrading the power transfer efficiency. In addition, as the power transfer system proposed in this study has a larger transmitting pad than the receiving pad, the misalignment causes little change in the self-inductance of the receiving coil, but the transmitting coil tends to increase the inductance. This is because the larger transmitting pad prevents a change in the magnitude of the magnetic field interlinked with the closed loop formed by the receiving coil, whereas the magnitude of the magnetic field interlinked with the closed loop formed by the transmitting coil increases owing to the opening of the area that was blocked by the receiving pad, as shown in Fig 3, which shows the magnetic flux distributions produced by simulation of the electromagnetic field produced under the alignment and misalignment conditions. The inductance of the transmitting coil increased in this manner shifts the resonance frequency to a lower frequency overall, which results in further degradation in the efficiency at the operating frequency that was used during alignment.

This problem can be solved by lowering the operating frequency to the shifted resonance frequency or by using a matching circuit [4, 29, 30] to shift the lowered resonance frequency to the original operating frequency and thus achieve the highest possible power transfer efficiency in the misalignment state. Even if such a compensation method succeeds in achieving the highest efficiency, it will still be lower than that realized during alignment. This is because misalignment reduces the mutual inductance. In this circumstance, an even larger transmitted current or power is needed to obtain the same level of received current or power as before. Such a larger current in the transmitting coil will further strengthen the magnetic fields around the power transfer system. Moreover, changes in the locations of the transmitting and receiving pads will also change the distributions of the magnetic fields. Normally, misalignment increases the magnetic field radiated from the transmitting coil so that the field can reach the more distant receiving coil. In summary, the following two main reasons produce a larger magnetic field in the case of misalignment than alignment.

1. Even if the same magnetic field is radiated from the transmitter, it is radiated more because the misalignment case produces a larger link path than the alignment.

2. If the received output falls due to misalignment, a stronger magnetic field is generated because a larger transmission output (current) is generated to compensate this.

Therefore, when considering the exposure scenario, it is preferable to consider the case of misalignment over the case of alignment.

### Anatomical human model

**Fig 4:**
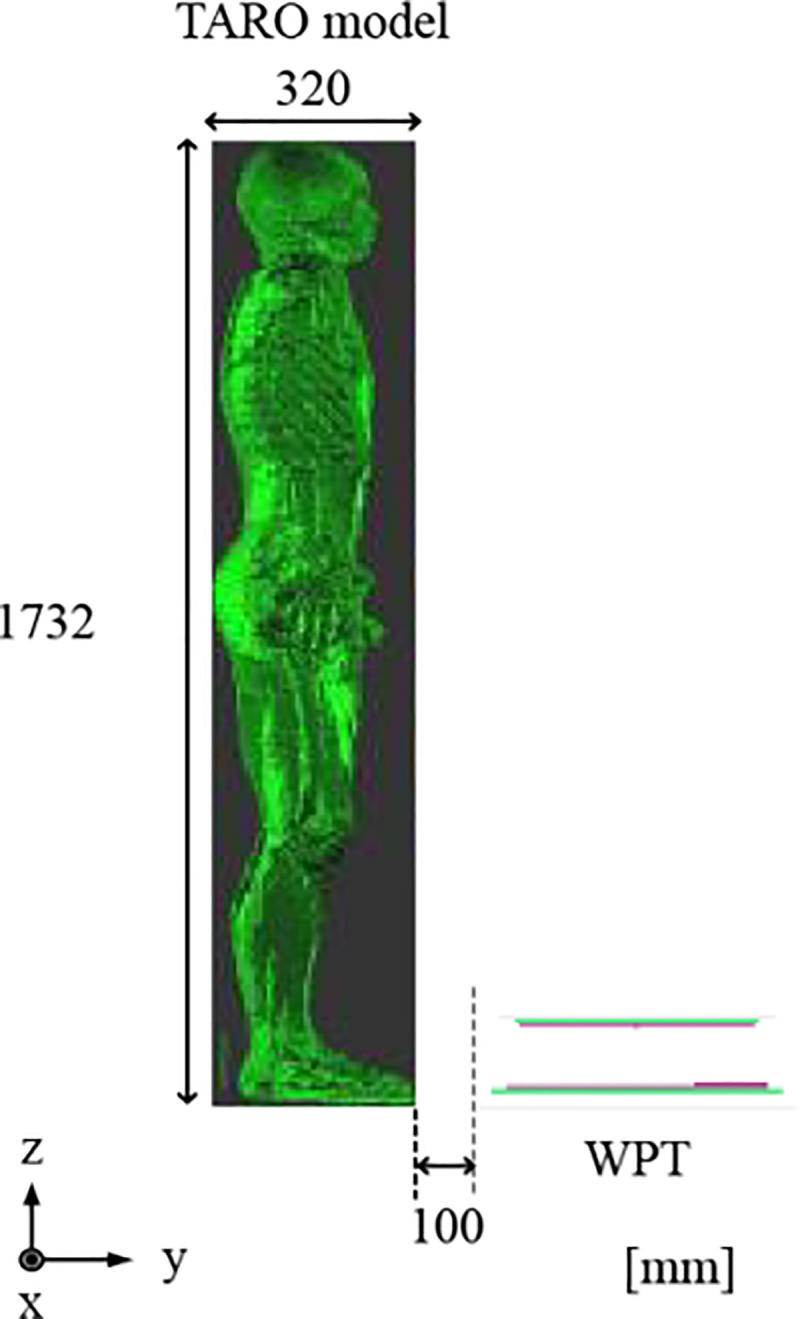
Anatomical whole-body voxel human model and position with respect to a WPT system (source: [33]).

In this study, a Japanese adult male whole-body voxel human model, TARO [31], is used to conduct dosimetry for a WPT system. TARO is an anatomical model of the human body developed on the basis of magnetic resonance imaging (MRI) data. The model possesses a spatial resolution of 2 mm and 51 tissues and organs. To carry out an electromagnetic simulation, the conductivity and permittivity of each tissue and organ should be set at the frequency of interest. Thus, the electrical properties are taken from Gabriel’s Cole-Cole models [32] (see appendix). The space occupied by the standing TARO model and surrounding air is 640 mm × 320 mm × 1732 mm. The transmitting pad of a WPT system will be located on the ground, and the receiving pad will be mounted on the floor pan of a vehicle body. To reflect a realistic exposure scenario, we assume that TARO stands by a WPT-mounted vehicle. However, this study focused on an evaluation of the exposure to only the WPT system without a vehicle body. Thus, the exposure scenario in which the TARO model stands in front of the WPT system was only supposed in this study. The location of the human body when evaluating the exposure was assumed to be a distance of 100 mm from the transmitting pad of the WPT system, as shown in Fig 4.

### Fabricated WPT system

**Fig 5:**
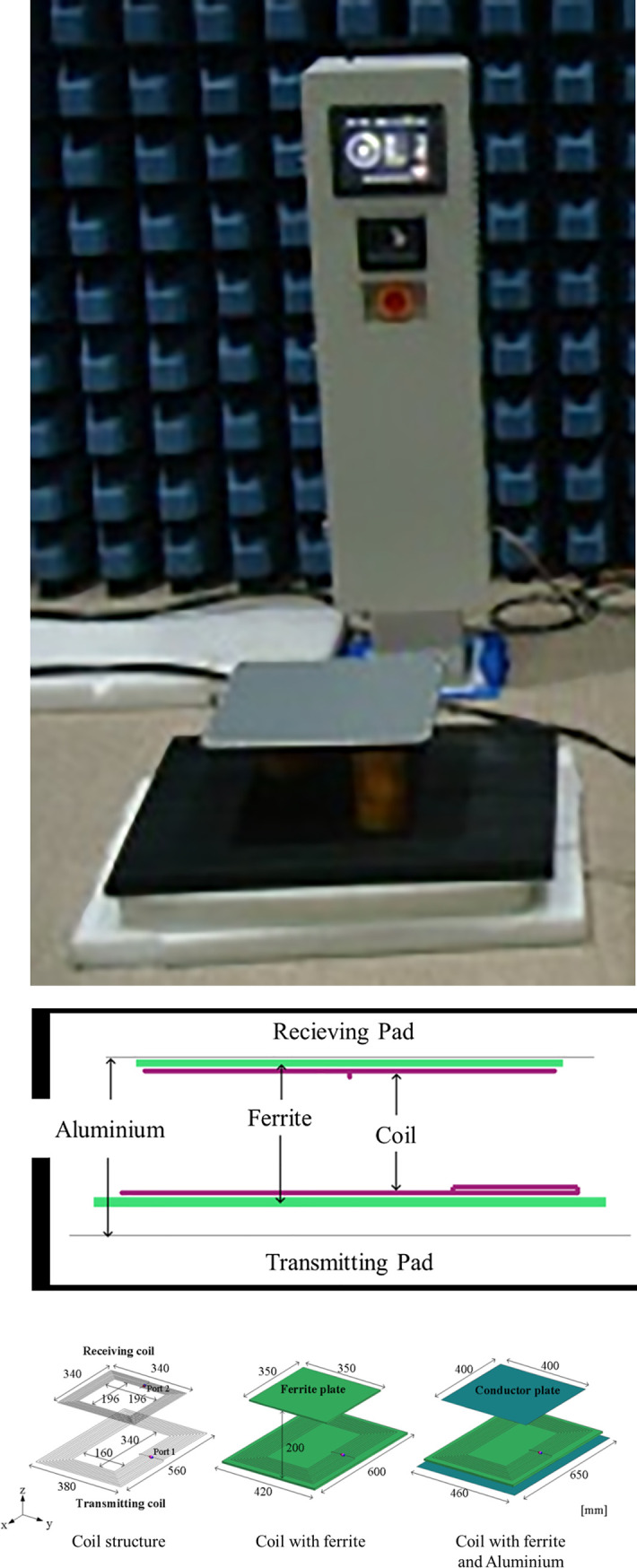
(a) Photograph of the developed WPT system for electric vehicles. (b) Side view of transmitting and receiving pad. (c) Coil, ferrite, and conductor plate arrangement of the WPT system (source: [33]).

In this study, a WPT system was designed to conform to the specifications [34] of a WPT system for electrical vehicles, which were provided by the Society of Automotive Engineers (SAE). Fig 5 shows the overall appearance of the developed system. The dimensions of the transmitting and receiving pads are 640 mm × 460 mm × 55 mm and 400 mm × 400 mm × 20 mm, respectively, as shown in Fig 5(C).

In this system, the receiving coil that is mounted on a vehicle has been designed to be smaller than the transmitting coil installed on the ground in order to compensate for the reduced power transfer efficiency during parking, which is caused by the misalignment between the transmitting and receiving coils. The numbers of turns of the transmitting and receiving coils are 12 and 13, respectively. The equivalent inductances were measured to be 135.19 and 120.13 μH (under alignment) for the transmitting and receiving coils, respectively. As shown in Fig 5(B), an aluminum plate is placed at the outermost part of a coil to provide shielding against electromagnetic waves, and a ferrite sheet is placed between a coil and the aluminum plate to prevent a drastic decrease in the efficiency due to eddy currents and to guide the electromagnetic fields. The clearance between the ground and the transmitting pad was set to 155 mm. This WPT system generates a voltage source (*V*_s_) between 81.38 kHz and 90 kHz at an inverter from an input single-phase voltage of 220 V at 60 Hz. The capacitances of the transmitting coil (*C*_1_) and receiving coil (*C*_2_) are set as 28.59 and 29.14 nF, respectively, to ensure that transmitting and receiving coils resonate at 81.38–90 kHz. The current flowing in the transmitting coil of the WPT system creates magnetic fields and induces another current (*I*_2_) in the receiving coil through electromagnetic induction. The crossing AC current is converted into a DC current (*I*_b_) by a rectifier and charges the battery of an electric vehicle. The WPT system designed in this study has a power transfer efficiency of approximately 90% [34]. The maximum output current (*I*_b_) and power were designed to be 20 A and 7 kW, respectively.

Until now, three variables affecting the change in the magnetic field have been discussed, and the fabricated WPT system has been described. The next section presents the process and the results obtained from experiments and simulations, which were performed to investigate the change in the magnetic field.

## Experiment and simulation

### Measurement

**Fig 6:**
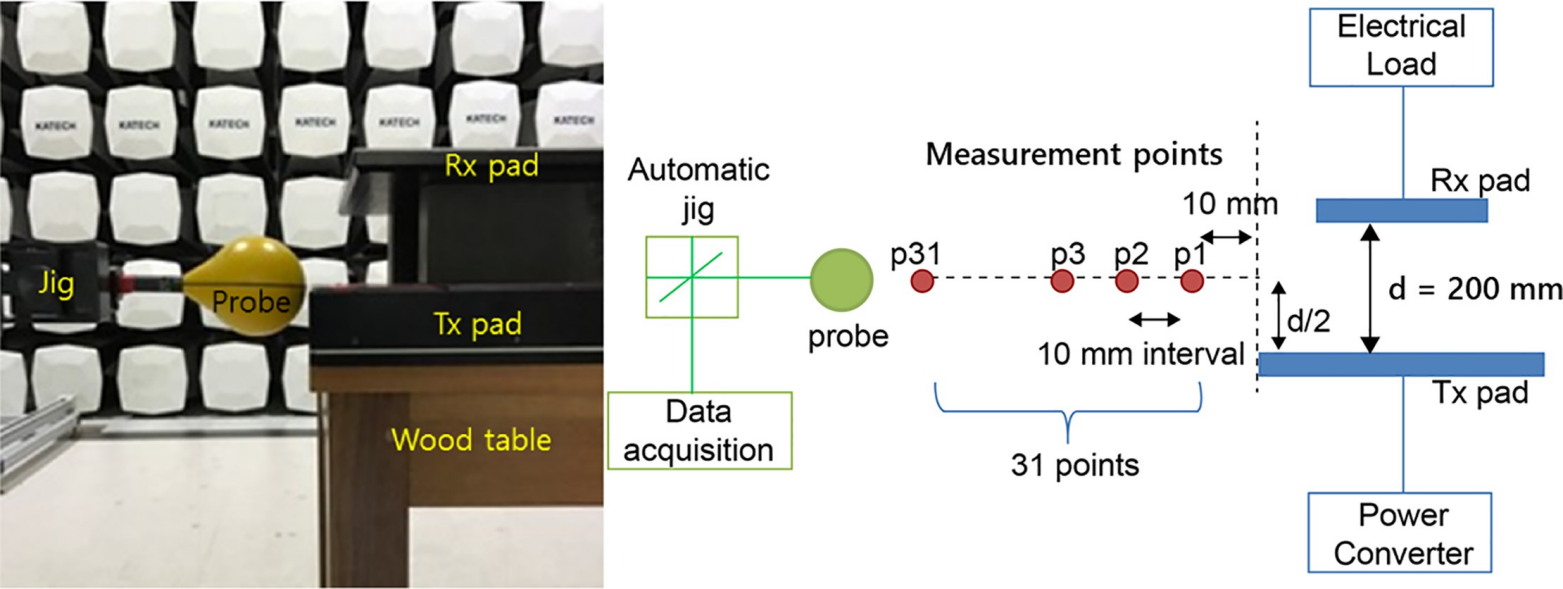
Measurement of the magnetic field around the WPT system using a magnetic field probe fixed to an automatic jig.

To measure the magnetic field around the WPT system, the transmitting pad was placed on a wooden table, and the receiving pad was separated from the transmitting pad by using a right-angled wood stick with a height of 100 mm. A probe (Narda, ELT 400) was used for measurement and was fixed by an automatic jig to ensure the exact measurement point. This probe can measure an external isotropic magnetic field. Fig 6 shows the experimental setup.

If measurement is performed by connecting the receiving end with a real battery, the battery is charged during the experiment, and the battery voltage changes, as mentioned above, thereby changing the current in the coil, which makes it difficult to obtain accurate measurements. In this study, this difficulty was overcome by replacing the battery with an electrical load (AMREL, PLW100K-600-600E). This DC electric load, which can operate in constant current, constant voltage, and constant power modes, has 100 kW, 600 V, and 600 A ratings. The proposed WPT system was designed to produce an output power as high as 7 kW. However, the output power for measuring a magnetic field was set to 3.3 kW to ensure the safety of the system.

The lithium-ion battery currently mounted on the electric vehicle is not fully charged or discharged in consideration of its life and damage. For example, it is specified that the batteries for electric vehicles maintain an SoC between 20% and 80%. This study also assumes that the SoC is maintained at this level. The electric vehicle used in this study was equipped with a lithium-ion-polymer battery designed to enable vehicle travel for approximately 180 km on a single charge. The battery has a capacity of 30 kWh and a rated voltage of 375 V. Although the situation may differ according to the type of battery for electric vehicles, measurement data were used to determine *V*_b_ = 340 V at an SoC of 20% and *V*_b_ = 380 V at an SoC of 80%. These values were determined by measuring the battery of the electric vehicle. When the desired value of *P*_out_ is determined, *I*_b_ to be supplied to the battery can be calculated by applying the current value of *V*_b_ and the formula *I*_b_ = *P*_out_/*V*_b_. *V*_S_ of the inverter in the transmitting end is adjusted to obtain *I*_b_ determined in this manner.

### Equivalent circuit model and simulation

As mentioned in Section I, a real WPT system consists of a resonance circuit part that relays energy, a circuit that generates a high-frequency source in the front end, and another circuit that stores energy in the rear end. This entire system should be considered to accurately simulate the nearby electromagnetic fields. A three-dimensional electromagnetic simulation using a finite-difference time-domain (FDTD) method and finite element method (FEM) is needed to analyze the electromagnetic fields of the transmitting and receiving coils. The remaining circuit parts can be analyzed by a circuit simulation such as the Simulation Program with Integrated Circuit Emphasis (SPICE). However, there is no perfect numerical analysis method that can cover both the circuit and electromagnetic simulations at once. Thus, as shown in Fig 2, the most convenient method is to perform an electromagnetic simulation of the resonance part with an equivalent circuit configuration, which is created by replacing the front-end circuit of the transmitting resonance part with *V*_S_ and the rear-end circuit of the receiving resonance part with *R*_L_. The latter operation is possible because the battery may be treated as a resistance in an instantaneous circumstance.

The electromagnetic analysis of the WPT system was performed by using FEKO [35], which is a commercial analysis tool developed on the basis of the method of moments (MoM). Since the entire system is difficult to simulate, as mentioned above, the most approximate simulation to the real system was implemented by setting *V*_S_ for the transmitting port and *R*_L_ for the receiving port. When the simulation conditions of *P*_out_ and *V*_b_ are determined, *I*_b_ is determined; then, *V*_S_ is adjusted to obtain the output current. In order to ensure the same simulation as the experimental conditions, *I*_2_ flowing in the receiving coil and *P*_out_ were measured in the experiment. *R*_L_ was obtained from the relation *R*_*L*_ = 2×*P*_*out*_/|*I*_2_|^2^; then, the simulation was carried out. If we want to simulate the electromagnetic field generated from a WPT system without an experiment, the current values of *V*_S_ and *I*_2_ can be determined by the results obtained from a circuit simulation of the WPT system.

### Comparison of the results

The experiment and simulation are aiming to examine the three variables that may cause a change in the electromagnetic fields of the WPT system. As for the charging mode, since the CV mode controls the voltage during the state of full charge and the power transfer for electric vehicle is stopped before the SoC reaches 100%, this mode was not considered. Because the maximum output power is specified as 3.3 kW and the battery voltage reaches its maximum value of 380 V in the CC mode, constant current transfer is conducted in this mode with an output current *I*_b_ of 8.7 A (3.3 kW/380 V). If the battery voltage is the lowest value of 340 V, the output power becomes 2.96 kW. In the CP mode, when the maximum output power is determined to be 3.3 kW, the highest battery voltage of 380 V creates the same conditions as the CC mode, whereas the lowest voltage of 340 V results in an output current of about 9.7 A. As the battery starts to be charged from 340 V to 380 V in the CP mode, the output current is gradually reduced from 9.7 A to 8.7 A. For this reason, the CC and CP modes can be compared with each other at a battery voltage of 340 V. As the battery voltage of 340 V is maintained as the voltage (*V*_2_) of the receiving end, the two modes have a similar value of the current (*I*_1_) in the transmitting coil. However, the CC mode maintains *I*_b_ at 8.7 A, whereas the CP mode has a higher value of *I*_b_ of 9.7 A. Both the experiment and simulation indicate that the CP mode has a slightly larger magnitude for the magnetic field than the CC mode for a battery voltage of 340 V. For example, the magnitudes of the magnetic field strength from the measurements in the CC and CP modes at point p1 in Fig 6 are 143.69 and 145.22 A/m, respectively. Similarly, those from the simulations in the CC and CP modes are 148.21 and 146.65 A/m, respectively. To compare the three variables affecting the electromagnetic radiation, the experimental and simulation results for three cases, which are presented in Table 1, are presented in this section.

**Table 1:** Three WPT system cases for investigating produced electromagnetic radiation strength.

Cases	Battery voltage (V)	Alignment between transmitting and receiving coils
1	340	Alignment^a^
2	380	Alignment
3	380	Misalignment^b^

Although a total of four combinations according to two charging states (20% SoC, 340 V and 80% SoC, 380 V) and two alignment states (alignment and misalignment) were considered, in this paper, only the above three cases except one case (20% SoC, 340 V and misalignment condition) are described for the sake of clarity and to avoid overlapping observations of misaligned characteristics.

^a^Transmitting and receiving pads’ centers are aligned

^b^The receiving pad has moved 75 mm to the front or back of or to the right or left of the its position with respect to the alignment state

**Fig 7:**
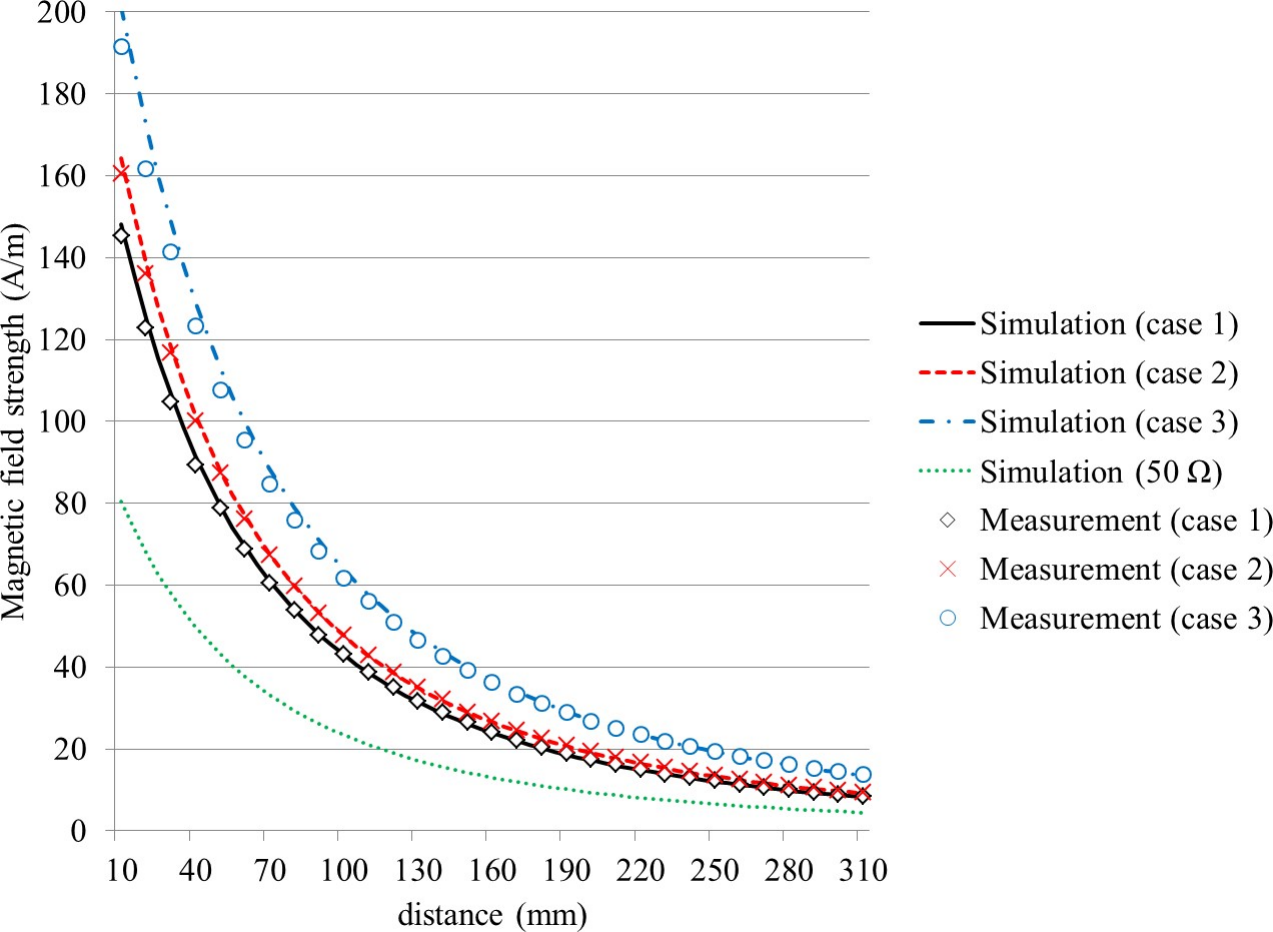
Comparison of the results obtained by measurement and simulation for the three cases, and the simulation results with a 50-Ω impedance at the terminal. The value on the x-axis means the distance between the Tx pad and the center of the probe.

The measurement was performed with 10-mm intervals at distances between 10 mm and 310 mm from the centers of the transmitting and receiving pads. Fig 7 shows the magnitudes of the magnetic field that were either measured by the probe or calculated by the simulation for the three operating conditions presented in Table 1.

The diameter of the probe was about 100 mm, and the measurements represented a magnetic field of that size. On the other hand, the simulated values corresponded to the central point where the probe was located. In addition, when the difference between the structural shape of the real WPT system and that used in the simulation is considered, the measurements and simulation show considerable agreement. This indicates the validity of the proposed simulation method using an equivalent circuit model, which was developed to describe a real WPT system in this study. Furthermore, another simulation was performed by setting the source and load to simply 50 Ω and applying 3.3 kW, which were the same conditions used in existing studies. The results are also displayed in Fig 7. As a result, the termination condition of 50 Ω caused a large difference in the magnetic field around the WPT system since the current flowing in the coils fundamentally changes, despite the same output of 3.3 kW. From the results of the three experiments and simulation, a higher battery voltage causes larger magnetic fields to be radiated, and misalignment causes a larger magnetic field to be radiated than that for alignment. This tendency can be confirmed by both the measurement and simulation. At a distance of 10 mm from the transmitting pad, the strength of the magnetic field in case 2 was 1.1 times that of case 1, and case 3 exhibited a 1.4 times stronger magnetic field than that of case 1. In addition, the values of self inductance and mutual inductance are shown in Table 2 for the validity of the experiment and simulation. Note that the values of the simulations extracted the equivalent parameters at the operating frequency. Considering the difference between the coils for simulation and the actual fabricated coils, we can see that they are almost identical.

**Table 2:** Inductances of measurement and simulation under alignment and misalignment.

	Measurement			Simulation		
	L_1_	L_2_	M	L_1_	L_2_	M
Alignment	135.19 μH	120.13 μH	29.18 μH	139.45 μH	123.67 μH	38.43 μH
Misalignment	138.48 μH	119.93 μH	22.85 μH	141.97 μH	123.54 μH	31.33 μH

## Guidelines and dosimetry

### ICNIRP guidelines

ICNIRP which is independent of any commercial, national, or otherwise vested interests, provides scientific advice and guidance on the health and environmental effects of non-ionizing radiation to protect people and the environment from detrimental electromagnetic radiation exposure. The ICNIRP limits, which are determined by various experts, are generally more stringent than the IEEE safety standard and, in most countries, are preferentially applied. The results of this paper are therefore discussed with respect to ICNIRP limits. As for the biological effects of an incident electromagnetic field, thermal effects dominate at a high frequency, whereas stimulation effects are prominent at a low frequency. ICNIRP guidelines provide the SAR (σ|*E*|^2^/*ρ*) as a basic restriction for protection against thermal effects in the frequency band of 100 kHz or more. For the frequency band of 10 MHz or below, the ICNIRP guidelines published in 1998 provide the current density (J) for protection against stimulation effects, and the revised ICNIRP guidelines published in 2010 provide limits based on the electric field of the 99^th^ percentile value (E99), which is the induced electric field as a vector average of the electric field in a small contiguous tissue volume of 2 x 2 x 2 mm^3^. Since the measurement or calculation of the induced quantities in a human body requires a considerable amount of technical effort, a reference level has been provided to measure and compare the electric and magnetic fields at the places where a human body will be positioned. The proposed WPT system has an operating frequency of 81.38–90 kHz, and Table 3 presents the limits of the ICNIRP guidelines, which correspond to this frequency band.

**Table 3:** ICNIRP exposure limits relevant for the WPT operating frequency.

Quantity	Frequency Range	Spatial Average	Value	ICNIRP
Basic Restrictions				
J	1–100 kHz	1 cm^2^ (square)	f/500 A/m^2^	1998
E99	3 kHz-10 MHz	2x2x2 mm^3^ (cube)	1.35x10-4 V/m	2010
Reference Levels				
E-field strength	3–150 kHz	point	87 V/m	1998
H-field strength			5 A/m	
B-field			6.25 μT	
E-field strength	3 kHz -10 MHz		83 V/m	2010
H-field strength			21 A/m	
B-field			27 μT	

f is in Hz

#### Numerical dosimetry

**Fig 8:**
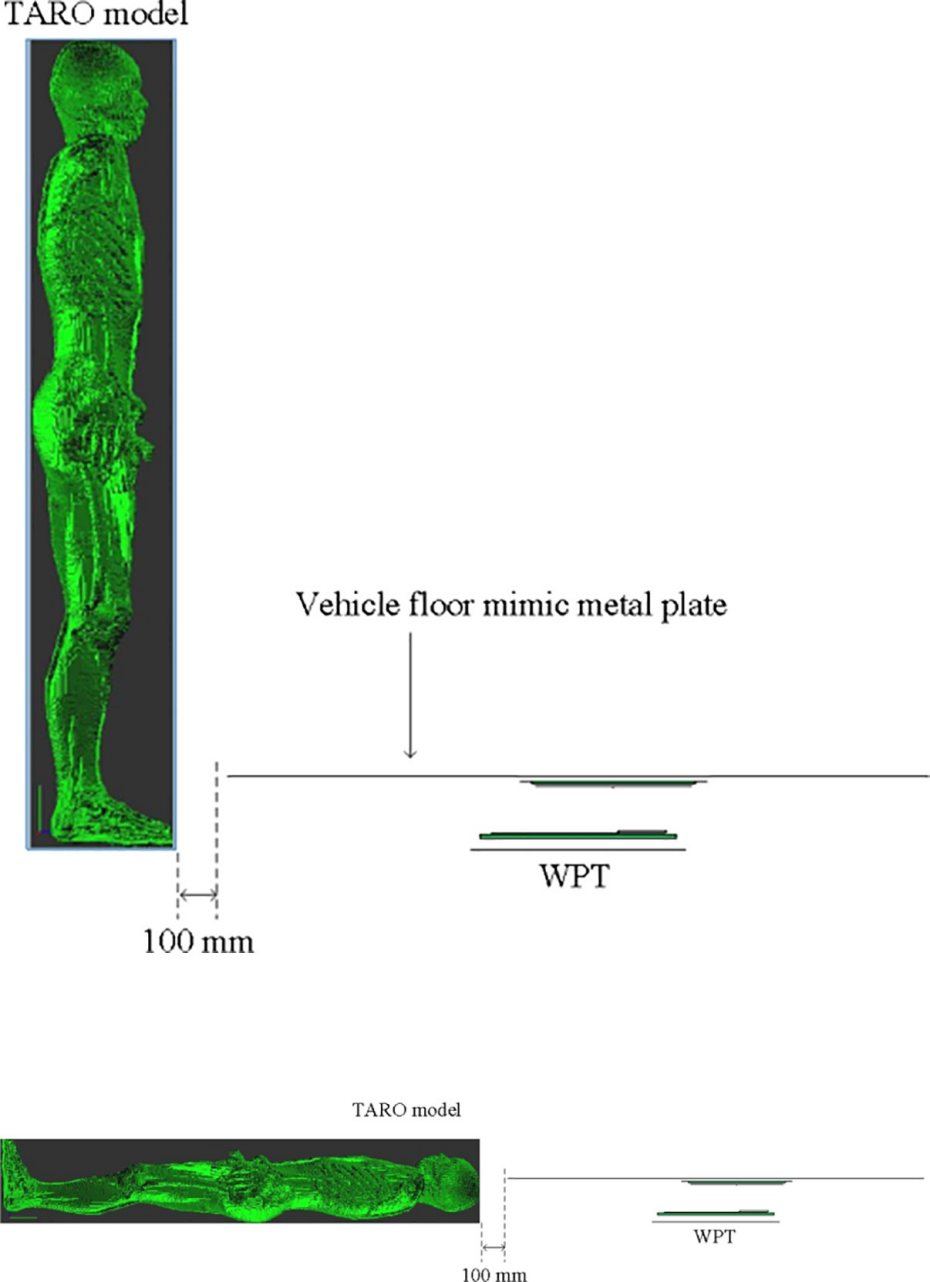
Anatomical whole-body voxel human model and position with respect to a WPT system with a vehicle floor-mimic metal plate for (a) standing exposure scenario (case 4) and (b) lying exposure scenario (case 5) (source: [33]).

**Fig 9:**
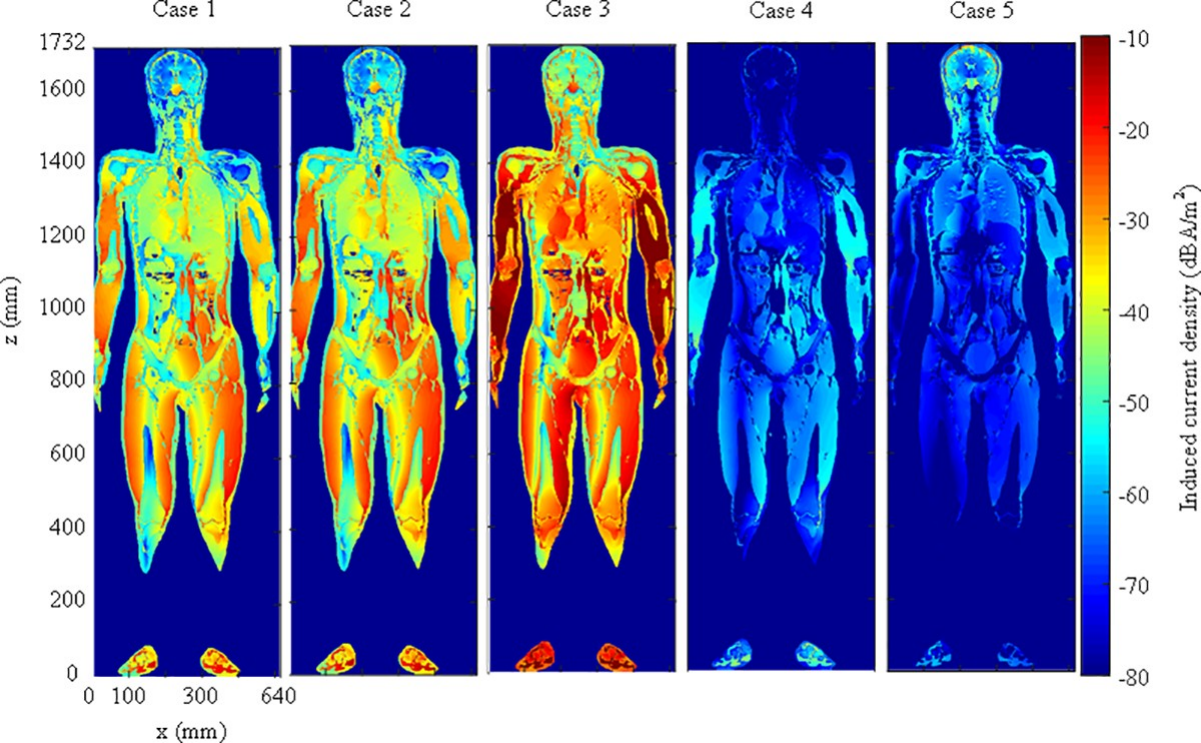
Cross-sectional current-density distributions for the five cases (cases 4 and 5 which include a conductor plate).

**Fig 10:**
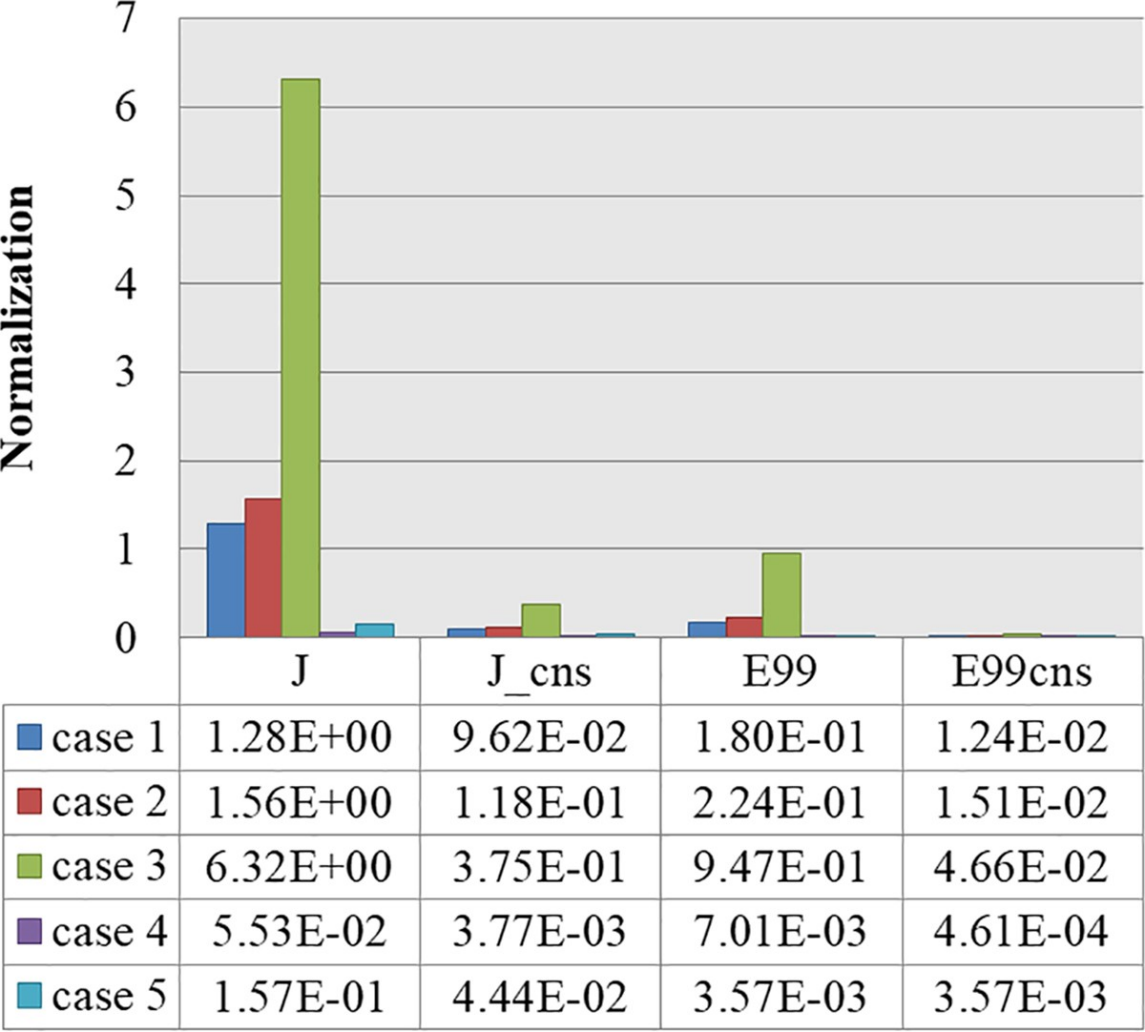
Induced quantities normalized by basic restrictions.

TARO was used to calculate the induced quantities in a human body for three cases (see Table 1). The calculation consisted of two stages [11, 14]. First, a simulation was performed to calculate the magnetic field at which a human body would be located near the WPT system. The magnetic field was regarded as the incident magnetic field to the human body, and the impedance method [36] (See appendix) was used to calculate the induced quantities. The prerequisite for this two stages technique is that the electromagnetic interaction between the wireless charging system and the human body can be negligible. This is because the amount of electromagnetic fields reflected by the human body and returned to the wireless charging system is very small. The verification has been done in previous studies [12, 14]. As the ratio of the electric field to the magnetic field of the space where the human body would be positioned was 5.6 Ω or below, the incident electric field could be neglected, as demonstrated in [14, 37]. Thus, only the quantities induced by the incident magnetic field were calculated in this study. Table 4 presents the results for the induced quantities for five cases: the three basic cases (case 1, case 2, and case 3, as shown in Table 1), and two additional cases (Fig 8) assuming exposure scenarios involving standing (case 4) and lying down (case 5) in front of a vehicle (the misalignment and SoC of 80% in cases 4 and 5 are equivalent to those in the case 3 condition: see Table 1). The exposure scenarios of cases 4 and 5 employ a 1-mm-thick, 1.5 m × 1.5 m metal plate as a vehicle mimic floor pan, as recommended in the SAE J2954, placed above the receiving pad. Fig 9 shows the current distributions of a cross section of the human body. In cases 1 to 4, J, E99, and Jcns & E99cns were the largest for the muscle, skin, and nerve spinal cord, respectively, while in case 5, the largest values of J and all other parameters were found in the CSF and grey matter, respectively. As was expected from the magnitudes of the magnetic field in each case, the induced quantity was the largest for case 3 followed by those for cases 2 and 1. In comparison with case 1, the induced quantity for case 2 was 1.2 times higher, and that for case 3 was about 5 times higher. In cases 4 and 5, in which the metal vehicle-mimic plate increases the body’s distance from the wireless charging system, the induced currents are reduced with respect to case 3 by factors of 0.0087 and 0.0248, respectively. Within the operating frequency range of the proposed system, as stated in Table 3, the reference level for a magnetic field was specified as 5 A/m in the 1998 version and 21 A/m in the 2010 version. The simulation based on the worst exposure scenario, which is case 3, for the WPT system with an output power of 3.3 kW showed that the human body should be located at a distance of 432 mm or more to comply with the 1998 guideline and 178 mm or more to conform to the 2010 guideline. As for the basic restrictions, J has a value of 162.76–180 mA/m^2^, and both E99 and E99_cns have a value of 10.9863–12.15 V/m in accordance with the frequency. The system proposed in this study was operated at 87.5 and 85 kHz for alignment and misalignment, respectively, to ensure the highest power transfer efficiency. At 87.5 kHz, the values of J and E99 were 175 mA/m^2^ and 11.8125 V/m, respectively. At 85 kHz, the values of J and E99 were 170 mA/m^2^ and 11.475 V/m, respectively. Fig 10 shows the normalized values of the induced quantities for the exposure scenarios by using the basic restrictions. As a result, only J does not satisfy the limit of the guidelines in the three basic scenarios although all limits including J are satisfied under the two additional scenarios involving the vehicle floor, and the revised 2010 version relaxed the limit of the 1998 guideline.

**Table 4:** Induced quantities in the human body for the three cases.

Case	J (A/m2)	Jcns (A/m2)	E99 (V/m)	E99cns (V/m)
1	2.241e-1	1.684e-2	2.128	1.462e-1
2	2.732e-1	2.062e-2	2.642	1.780e-1
3	1.074	6.375e-2	1.086e+1	5.346e-1
4	9.411e-3	6.416e-4	8.043e-2	5.288e-3
5	2.664e-2	7.549e-3	4.099e-2	4.099e-2

J is averaged over a 1-cm^2^ cross section perpendicular to the current direction (A/m^2^), which is used in the ICNIRP guidelines published in 1998.

Jcns is J in central nervous system (CNS) tissue (A/m^2^).

E99 is the 99^th^ percentile value of the electric field (V/m), which is from the ICNIRP guidelines published in 2010.

E99cns is the 99^th^ percentile value of the electric field in CNS tissue (V/m).

It should be noted that the accuracy issue of the evaluation with the metric, E99, has been mentioned by international standardization [38].

## Conclusions

This study proposed an equivalent circuit model method to represent the source and load conditions for an accurate description of the magnetic field formed around a WPT system. The effectiveness of the method was experimentally verified. In addition, three core variables (the charging mode, the SoC, alignment or misalignment between the transmitting and receiving coils) were also examined. They are factors that influence the strength of the magnetic field generated near an operating WPT system. As for the charging mode, the CP mode distributed the magnetic field slightly more intensively than the CC mode. At a distance of 10 mm from the transmitting pad of the system, when the most typical CC mode was assumed to be applied for power transfer, the SoC of 80% (380 V) produced a magnetic field that is 1.1 times stronger than that at an SoC of 20% (340 V), and within the conditions of an SoC of 80% (380 V), misalignment produced a magnetic field that is 1.27 times stronger than that during alignment. Needless to say, these results will change according to the specifications of the batteries mounted in electric vehicles. Under the assumption that the same power is transmitted, a lower rated voltage of the battery will cause a larger current, and a larger magnetic field will be generated. According to other resonance topologies, the current flowing in the coil will also change. The results of this study indicate that the worst exposure scenario occurs when the transmitting and receiving coils are misaligned and the battery is nearly fully charged. The importance of the potential implications of the exposure evaluation of WPT systems is in the order of misalignment, the SoC, and the charging mode. In the worst exposure scenario of this study that the wireless charging system with a 3.3 kW output is almost fully charged and is 75 mm misaligned, when the human body was standing at a point 100 m away from the charging pad, it was 6.32 times higher than the basic restriction of ICNIRP 1998. In future work, dosimetry will be conducted for a WPT-mounted vehicle and WPT systems having other resonance topologies.

## Appendix

The electrical properties of human body tissues at 85 kHz used in this study from the parametric model and the parameter values [34] developed by C. Gabriel based on their measurements are shown in Table 5. The analytical space of TARO model with 2 mm spatial resolution is 320 × 160 × 866 (x × y × z). The impedance for each voxel can be expressed as the following equation with the conductivity and relative permittivity presented in Table 5.

**Table 5:** Conductivity and relative permittivity of biological tissues at 85 kHz for adult male model.

Tissue name	σ(s/m)	*ε*_*r*_	Tissue name	σ(s/m)	*ε*_*r*_	Tissue name	σ(s/m)	*ε*_*r*_
Cerebellum	0.152	3923	Duodenum	0.535	2988	Thymus gland	0.536	3439
CSF	2.000	109	Esophagus	0.535	2988	Thyroid gland	0.536	3439
Cornea	0.495	11710	Gall bladder bile	1.400	120	Trachea	0.336	4298
Dura	0.502	339	Gall bladder	0.900	109	Testicles	0.359	8497
Vitreous humour	1.500	98	Heart	0.210	11140	Blood	0.702	5145
Grey matter	0.132	3629	Kidney	0.168	8352	Cortical bone	0.021	235
hypothalamus	0.106	3003	Liver	0.081	8132	Cancellous bone	0.044	311
Lens	0.340	2166	Lung	0.188	4326	Cartilage	0.178	2613
Pineal body	0.106	3003	pancreas	0.359	8497	Fat	0.024	106
Pituitary gland	0.106	3003	Testis prostate	0.437	5870	Muscle	0.359	8497
Salivary gland	0.359	8497	Small intestine	0.590	14520	Spinal chord	0.078	5923
Thalamus	0.106	3003	SIC	0.359	8497	Skin	0.028	9061
Tongue	0.287	4893	Spleen	0.121	4462	Tooth	0.021	235
White Matter	0.081	2376	Stomach	0.535	2988	Bladder	0.218	1356
Adrenal gland	0.359	8497	Stomach content	0.359	8497	Tendon	0.388	531
Large intestine	0.247	3888	Phren	0.359	8497	Seminal vesicle	0.359	8497
LIC	0.359	8497	Urina	0.702	5145	Corpus spongiosum	0.359	8497

CSF: Cerebral spinal fluid, LIC: Large intestine content, SIC: Small intestine content

In this paper, the skin conductivity proposed by Gabriel’s model is used as it is. However, recently, a skin conductivity model has been proposed by De Santis et al [40]. It should be noted that the skin conductivity value could affect the simulation results.

$$Z=\frac{l}{(\sigma +j\omega \varepsilon_{0}\varepsilon_{r})S}$$

where *σ* and *ε*_*r*_ represent the conductivity and relative permittivity of the human body tissue, *ε*_0_ is the permittivity of free space, and l and *S* represent the length of the edge and the cross-sectional area of a voxel. An inhomogeneous human body can be modeled as a three-dimensional impedance network by calculating the x, y, and z directed impedances along each voxel edge in the impedance method [36]. The closed loop voltage (electromotive force) in a cross-sectional area induced by incident magnetic field can be found from Faraday’s law as

$$V(x,y,z)=-\frac{\partial }{\partial t}\iint \mu_{0}\vec{H}(x,y,z)∙\vec{ds}$$

where *ds* is a unit of the area within the loop and *μ*_0_ is the permeability of free space. All loop currents in the impedance network of human body can be obtained by solving the linear system of equations with the iteration method of successive overrelaxation algorithm [39]. The internal electric field is given by

$$\vec{E}=\frac{\vec{J}}{\sigma +j\omega \varepsilon_{0}\varepsilon_{r}}$$

where *J* represents the current density.
